# MOLECULAR DETERMINANTS OF POOR T CELL MEMORY DURABILITY IN OLDER ADULTS

**DOI:** 10.1093/geroni/igae098.1288

**Published:** 2024-12-31

**Authors:** Ines Sturmlechner, Abhinav Jain, Jorg Goronzy

**Affiliations:** Department of Immunology, Mayo Clinic, Rochester, Minnesota, United States; Mayo Clinic; Mayo Clinic

## Abstract

Generation of a protective and long-lasting immune memory is central to effective vaccination strategies and healthy aging. However, age-associated changes in immune cells, particularly T cells, impair immune memory generation and maintenance rendering older adults vulnerable to infectious diseases. Here, we investigated signatures of age-related memory T cell dysfunction and uncovered molecular determinants of a protective, persistent T cell memory in older individuals. We leveraged the contrasting T cell memory elicited in younger (<30 years) and older adults (>55 years) by vaccination against varicella zoster virus (VZV). Using ex vivo peptide stimulation of peripheral blood mononuclear cells of vaccine recipients several years after vaccination, we collected antigen-specific CD4+ and CD8+ T cells for high-dimensional spectral flow cytometry and tri-modal single-cell sequencing analyses. We found that older vaccine recipients predominantly suffer from a quantitatively and qualitatively impaired CD8+ T cell memory response compared to younger adults receiving the same vaccine type. Conversely, comparisons of two VZV vaccine strategies with contrasting efficacies in older adults demonstrated that a CD4+ T cell response rich in self-renewing Th17-like and low in regulatory T cells overcomes the age-associated T cell memory defect and associates with a durable, protective T cell memory. Collectively, we propose that vaccine strategies targeted towards older adults benefit from a Th17-focused CD4+ T cell memory response. Our results not only uncover traits of immune cell dysfunction with aging, but also inform vaccination efforts tailored towards healthy aging.

